# Supplementary material for: A next-generation anti-CTLA-4 probody mitigates toxicity and enhances anti-tumor immunity in mice
Source: Nat Commun. 2025 Oct 10;16:9029. doi: 10.1038/s41467-025-64081-y (PMC12514261; doi:10.1038/s41467-025-64081-y)
Supplement: Supplementary file 1 — Supplementary Information [file 41467_2025_64081_MOESM1_ESM.pdf]

Supplementary Information for

A next-generation anti-CTLA-4 probody mitigates toxicity and enhances anti-tumor immunity in mice

Weian Cao[1,2#], Junfan Chen[1,2#], Yutong Fu[1,2#], Haitao Jiang[1,2], Yu Gao[1,2], Huiming Huang[1,2], Yang-Xin Fu[1,2,3]*, Wenyan Wang[1,2]*

[1]School of Basic Medical Sciences, Tsinghua University, Beijing 100084, China

[2]State Key Laboratory of Molecular Oncology, School of Basic Medical Sciences, Tsinghua University, Beijing 100084, China

[3]Changping Laboratory, Changping District, Beijing, China

[#]These authors contributed equally to this work.

*Corresponding author: wywang2022@tsinghua.edu.cn, yangxinfu@tsinghua.edu.cn

Supplementary information introduces:

Supplementary Figures. 1 to 5

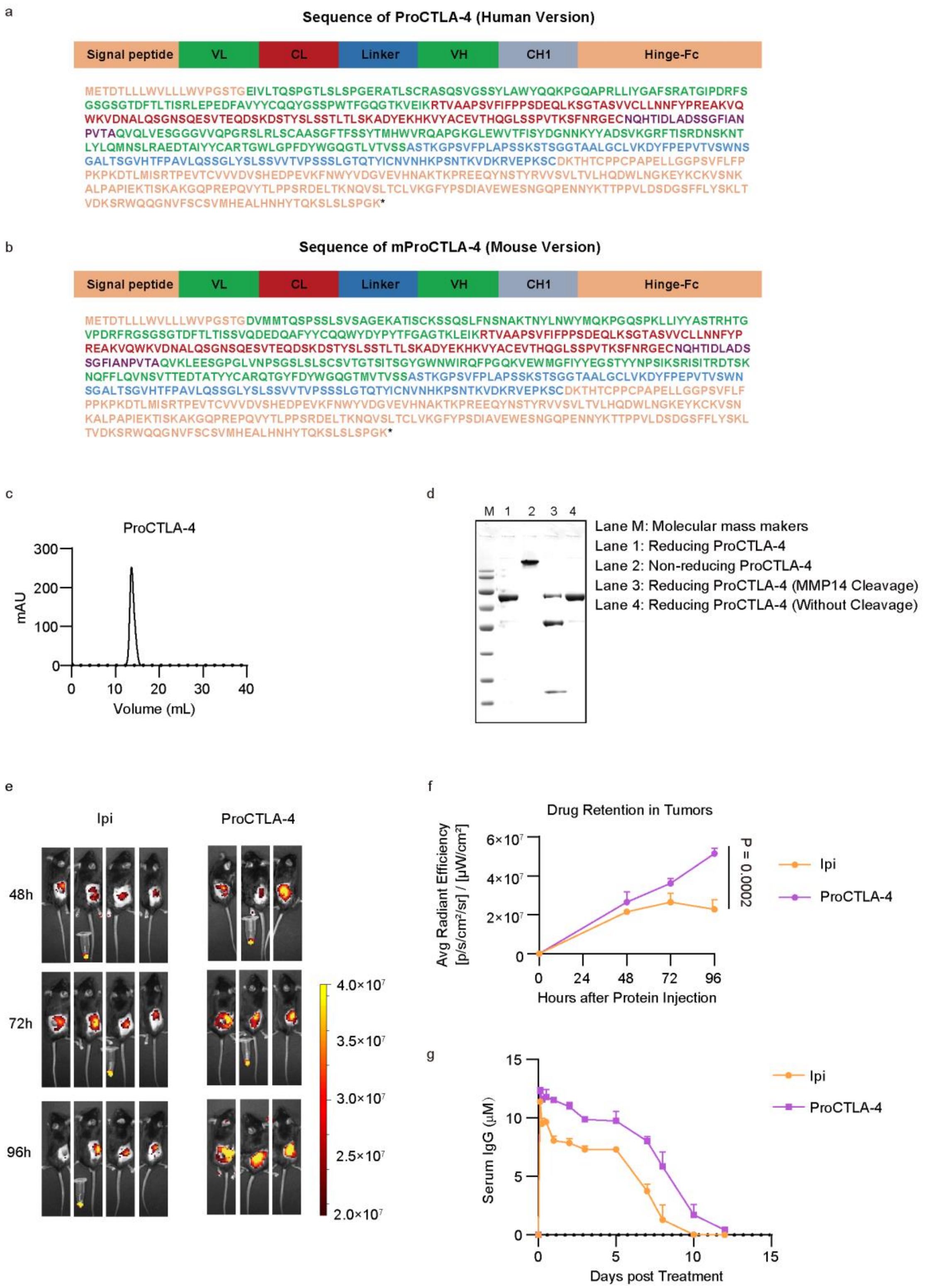

Supplementary Figure 1. The distribution of ProCTLA-4.

The sequence and motifs of human (**a**) and mouse (**b**) ProCTLA-4 are shown. **c,** The purity of purified ProCTLA-4 was analyzed by size exclusion chromatography. **d**, 2 μg purified ProCTLA-4 was individually incubated with or without MMP14 at 37 °C

overnight. The MMP14-digested ProCTLA-4 under different conditions was analyzed by SDS-PAGE. **e,f,** MC38 tumor-bearing male human CTLA-4 knocked-in C57BL/6 mice (hCTLA-4 KI mice) were injected i.v. with 40 μg cy5-labelled Ipilimumab (Ipi-cy5) (n=4) or equimolar cy5-labelled ProCTLA-4 (ProCTLA-4-cy5) (n=3) on day 11 after tumor inoculation. The accumulation of individual proteins in tumors was detected by the in vivo imaging system at 48h, 72h, and 96h after injection. The image (**e**) and the quantification curve (**f**) were shown. **g**, Equimolar doses of ProCTLA-4 or Ipi (10 μg) (n=3/group) were injected i.p. into male hCTLA-4 KI mice, and serum was collected at indicated time points. hIgG ELISA was used to quantify the amount of ProCTLA-4 or Ipi in the serum. Data (**e-g**) are representative of two independent experiments. Statistical analysis was performed using two-way ANOVA test (**f**).

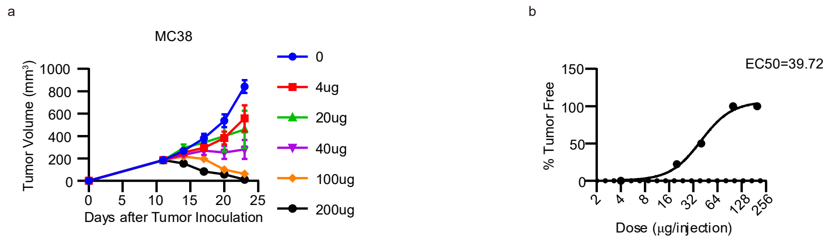

Supplementary Figure 2. The EC50 of ProCTLA-4 on MC38 tumor.

MC38 tumor-bearing male hCTLA-4 KI mice were i.p. treated with ProCTLA-4 at escalating doses (4 µg (n=4), 20 µg (n=4), 40 µg (n=8), 100 µg (n=4), or 200 µg (n=4)) on days 11, 14, and 17 after tumor inoculation. **a**, Tumor growth was monitored. **b**, Dose-dependent tumor-regression rates were analyzed to calculate the EC50 for complete tumor killing.

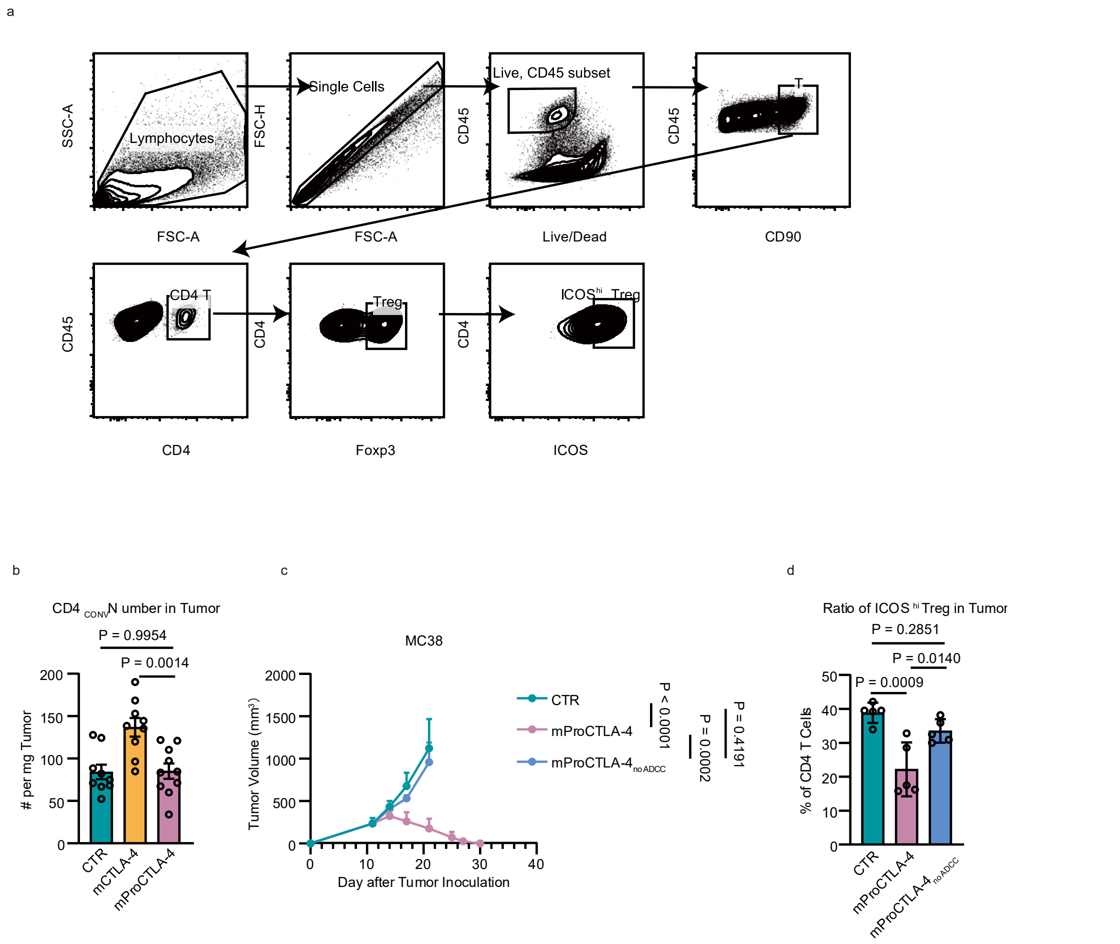

Supplementary Figure 3. The efficacy of ProCTLA-4 depends on FcγR engagement.

a, Gating strategy of Treg (L/D$^-$CD45$^+$CD90$^+$CD4$^+$Foxp3$^+$), CD4conv (L/D$^-$CD45$^+$ CD90$^+$CD4$^+$Foxp3$^-$), and ICOS^{hi} Treg (L/D$^-$CD45$^+$CD90$^+$CD4$^+$Foxp3$^+$ICOS^{high}). **b,** MC38 tumor-bearing male mice were treated i.p. with CTR (n=9), 10 µg mCTLA-4 (n=9), or equimolar mProCTLA-4 (n=10) on day 14 after tumor inoculation. 3 days later, the number conventional CD4$^+$ T cells in the tumor was analyzed. **c,** MC38 tumor-bearing male mice were treated i.p. with 50 µg mProCTLA-4, or equimolar mProCTLA-4-Fc with no ADCC function (mProCTLA-4$_{no\ ADCC}$) (n=5/group) on days 11, 14, and 17 after tumor inoculation. The tumor curve was monitored. **d,** MC38 tumor-bearing male mice were treated i.p. with PBS (CTR), 10 µg mProCTLA-4, or equimolar mProCTLA-4$_{no\ ADCC}$ (n=5/group) on day 14 after tumor inoculation. 2 days later, the ratio of ICOS^{hi} Treg in the tumor was analyzed. Data (**b-d**) are representative of two independent experiments. Statistical analysis was performed using ordinary one-

way ANOVA with multiple comparisons test (**b, d**) or two-way ANOVA test (**c**).

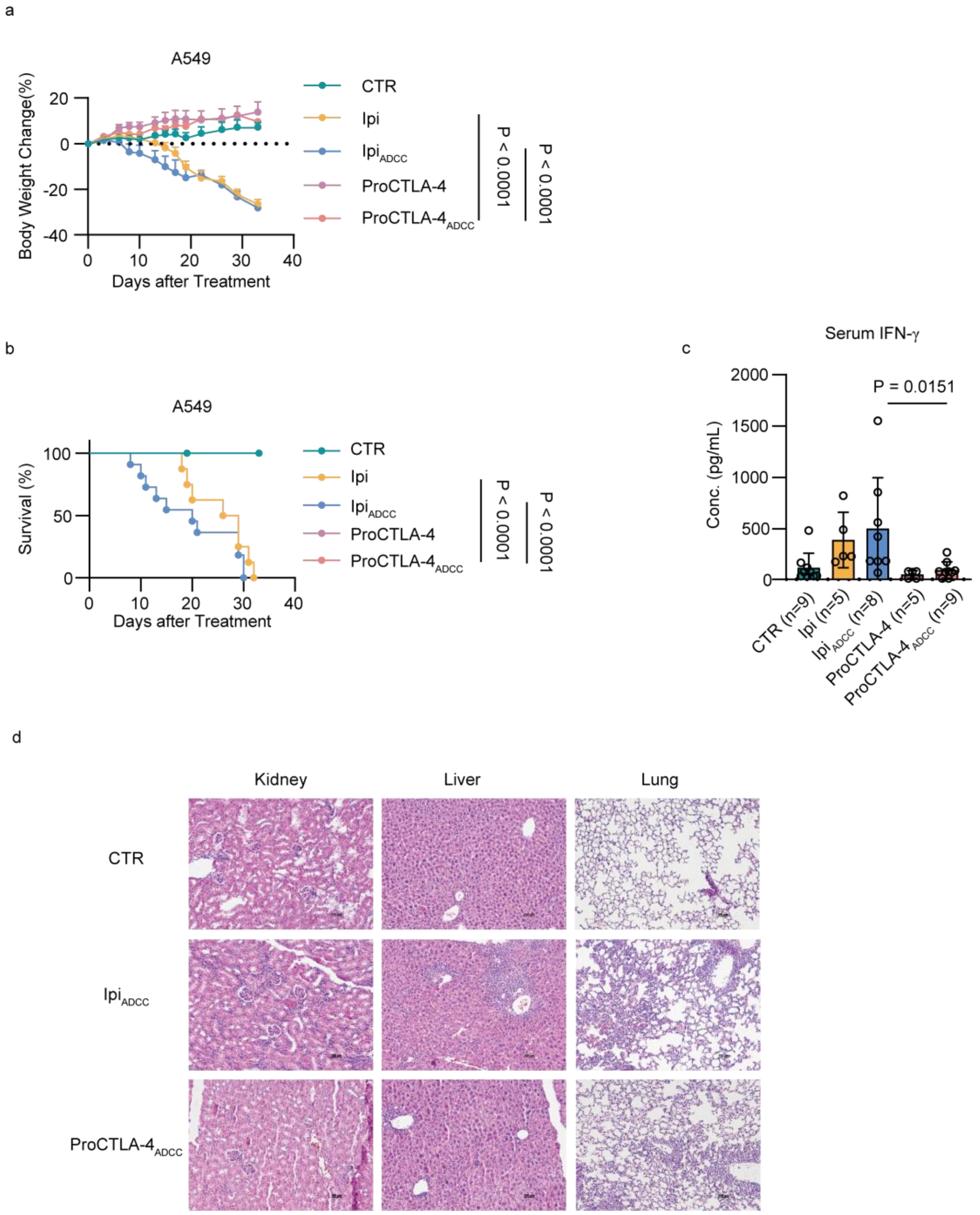

Supplementary Figure 4. The toxicity of ProCTLA-4 with ADCC enhanced Fc.

a-d A549 tumor-bearing male NSS mice were transferred 5 million human PBMCs i.v. on day 8 after tumor inoculation and were i.p. treated with PBS (CTR) (n=13), 200 µg Ipilimumab (Ipi) (n=8), equimolar ADCC-enhanced Ipilimumab (Ipi_{ADCC}) (n=11), ProCTLA-4 (n=9), or ADCC-enhanced ProCTLA-4 ($ProCTLA\text{-}4_{ADCC}$) (n=11) on days 10, 13, and 16 after tumor inoculation. The percentage of body weight change (**a**), the percentage of survival (**b**), the serum IFN-γ level at 24h after the third treatment (**c**), and the representative images of H&E stained paraffin sections from the kidney, liver

and lung were shown (**d**). Scale bar, 100 μm. Data are representative of two independent experiments. Statistical analysis was performed using two-way ANOVA with Tukey's multiple comparisons test (**a**), log-rank (Mantel-Cox) test (**b**), or ordinary one-way ANOVA with multiple comparisons test (**c**).

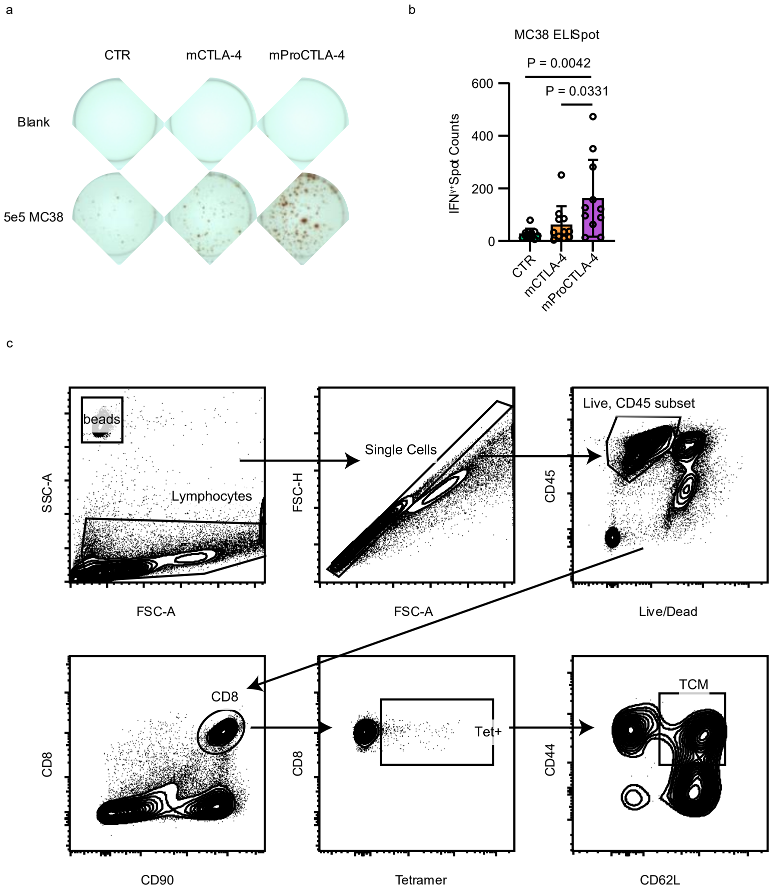

Supplementary Figure 5. The ELISpot assay of ProCTLA-4 treated MC38 tumor. a, b, MC38 tumor-bearing male mice were i.p. treated with CTR, 40 μg anti-mouse CTLA-4 antibody (mCTLA-4), or equimolar mProCTLA-4 (n=11/group) on days 10 and 13. Mice were euthanized at the endpoint and lymphocytes from dLN were collected for ELISpot assay. **c,** Gating strategy of Tetramer^{+} CD8^{+} central memory T cells (L/D^{-}CD45^{+}CD90^{+}CD8^{+}Tetramer^{+}CD44^{+}CD62L^{+}). Data are representative of two independent experiments. Statistical analysis was performed using ordinary one-way ANOVA with multiple comparisons test.
