# Supplementary material for: A next-generation anti-CTLA-4 probody mitigates toxicity and enhances anti-tumor immunity in mice
Source: Nat Commun. 2025 Oct 10;16:9029. doi: 10.1038/s41467-025-64081-y (PMC12514261; doi:10.1038/s41467-025-64081-y)
Supplement: Supplementary file 2 — Reporting Summary [file 41467_2025_64081_MOESM2_ESM.pdf]

Corresponding author(s): Yang-Xin Fu, Wenyan Wang

Last updated by author(s): Aug 20, 2025

Reporting Summary

Nature Portfolio wishes to improve the reproducibility of the work that we publish. This form provides structure for consistency and transparency in reporting. For further information on Nature Portfolio policies, see our Editorial Policies and the Editorial Policy Checklist.

Statistics

For all statistical analyses, confirm that the following items are present in the figure legend, table legend, main text, or Methods section.

n/a	Confirmed	
☐	☒	The exact sample size (*n*) for each experimental group/condition, given as a discrete number and unit of measurement
☐	☒	A statement on whether measurements were taken from distinct samples or whether the same sample was measured repeatedly
☐	☒	The statistical test(s) used AND whether they are one- or two-sided<br>*Only common tests should be described solely by name; describe more complex techniques in the Methods section.*
☒	☐	A description of all covariates tested
☒	☐	A description of any assumptions or corrections, such as tests of normality and adjustment for multiple comparisons
☐	☒	A full description of the statistical parameters including central tendency (e.g. means) or other basic estimates (e.g. regression coefficient) AND variation (e.g. standard deviation) or associated estimates of uncertainty (e.g. confidence intervals)
☐	☒	For null hypothesis testing, the test statistic (e.g. *F*, *t*, *r*) with confidence intervals, effect sizes, degrees of freedom and *P* value noted<br>*Give P values as exact values whenever suitable.*
☒	☐	For Bayesian analysis, information on the choice of priors and Markov chain Monte Carlo settings
☒	☐	For hierarchical and complex designs, identification of the appropriate level for tests and full reporting of outcomes
☒	☐	Estimates of effect sizes (e.g. Cohen's *d*, Pearson's *r*), indicating how they were calculated

Our web collection on statistics for biologists contains articles on many of the points above.

Software and code

Policy information about availability of computer code

Data collection	BD FACSDiva™ Software; IVIS LUMINA.
Data analysis	FlowJow (version 10); Graphpad Prism 10; Adobe Illustrator 2025; R(4.3.3); Cell Ranger (V.8.0.0); R package Seruat (V5.0.3); R Studio (V.2023.12.1); ggplot2 (V.3.5.1).

For manuscripts utilizing custom algorithms or software that are central to the research but not yet described in published literature, software must be made available to editors and reviewers. We strongly encourage code deposition in a community repository (e.g. GitHub). See the Nature Portfolio guidelines for submitting code & software for further information.

Data

Policy information about availability of data

All manuscripts must include a data availability statement. This statement should provide the following information, where applicable:

- Accession codes, unique identifiers, or web links for publicly available datasets
- A description of any restrictions on data availability
- For clinical datasets or third party data, please ensure that the statement adheres to our policy

All data that support the findings of this study are available from the corresponding author on reasonable request.

Research involving human participants, their data, or biological material

Policy information about studies with human participants or human data. See also policy information about sex, gender (identity/presentation), and sexual orientation and race, ethnicity and racism.

Reporting on sex and gender	No human participants is included.
Reporting on race, ethnicity, or other socially relevant groupings	No human participants is included.
Population characteristics	No human participants is included.
Recruitment	No human participants is included.
Ethics oversight	No human participants is included.

Note that full information on the approval of the study protocol must also be provided in the manuscript.

Field-specific reporting

Please select the one below that is the best fit for your research. If you are not sure, read the appropriate sections before making your selection.

☒ Life sciences ☐ Behavioural & social sciences ☐ Ecological, evolutionary & environmental sciences

For a reference copy of the document with all sections, see nature.com/documents/nr-reporting-summary-flat.pdf

Life sciences study design

All studies must disclose on these points even when the disclosure is negative.

Sample size	Sample sizes were predetermined from pilot experiments and/or experiments that have been done in the past, to obtain statistically significant data.
Data exclusions	No data were excluded from the analysis.
Replication	As reported in the figure legends, experiments were performed two or three times with similar results.
Randomization	For all in vivo experiments, animals were randomly assigned into a treatment group after tumor inoculation. The starting tumor burden in the treatment and control groups was similar before treatment. For all in vitro experiments, samples with the same origin were randomly allocated into control and experimental groups.
Blinding	Investigators were not blinded to group allocation during experimental setup, data collection, and analysis. No blinding was performed since we did not have the personnel resources to consistently perform blinding.

Reporting for specific materials, systems and methods

We require information from authors about some types of materials, experimental systems and methods used in many studies. Here, indicate whether each material, system or method listed is relevant to your study. If you are not sure if a list item applies to your research, read the appropriate section before selecting a response.

Materials & experimental systems

n/a	Involved in the study	
☐	☒	Antibodies
☐	☒	Eukaryotic cell lines
☒	☐	Palaeontology and archaeology
☐	☒	Animals and other organisms
☒	☐	Clinical data
☒	☐	Dual use research of concern
☒	☐	Plants

Methods

n/a	Involved in the study	
☒	☐	ChIP-seq
☐	☒	Flow cytometry
☒	☐	MRI-based neuroimaging

Antibodies

Antibodies used	For in vivo depletion: Anti-CD8 antibody (Lyt 3.2,BE0223) and anti-CD4 antibody (GK1.5,BE0003-1) were purchased from BioXcell.

For FACS:
Anti-mCD45 (30-F11), eBioscience, 47-0451-82;
Anti-mCD90.2 (Thy-1.2), Biolegend, 105306;
Anti-mCD90.2 (Thy-1.2), Biolegend, 105306;
Anti-mCD4(GK1.5), Biolegend, 100451;
Anti-mFoxp3(fjk-16s),eBioscience, 12-5773-82;
Anti-mCD8 （KT15), MBL, K0227-4;
T-Select H-2KbOVA Tetramer-SIINFEKL-PE, MBL, TS-5001-1c;
Anti-mCD44(IM7) Biolegend, 103026;
Anti-mCD62L(MEL14), Biolegend, 104428;
Anti-mCD4(GK1.5), Biolegend, 100451;
Anti-mFoxp3(fjk-16s),eBioscience, 12-5773-82;
Anti-mCD278(ICOS)(C398.4A) Biolegend, 313520;
Purified anti-mouse CD16/32 Antibody, Biolegend, 101302;
Fixable Viability Dye eFluor™ 506, BD, 65-0866-14.
For Cytometric Bead Array: anti-mIFN-γ and anti-mTNF, BD Cytometric Bead Array mouse inflammation Kit, 552364; anti-hIFN-γ, BD Cytometric Bead Array human TH1/TH2/TH17 CBA Kit, 560484.

Validation

All antibodies were well-recognized clones in the field and validated by the manufacturer websites for validation data. These antibodies are routinely used in our laboratory without additional validation.

Eukaryotic cell lines

Policy information about cell lines and Sex and Gender in Research

Cell line source(s)	B16F10, CT26 and A549 cell lines were purchased from the American Type Culture Collection. MC38 was purchased from the Cytion. Freestyle 293 F cell (R79007) was purchased from Invitrogen.
Authentication	The cell lines from manufacturers were not authenticated. The MC38-hCTLA-4 cell line was authenticated by flow cytometry.
Mycoplasma contamination	All cell lines were tested negative for the presence of mycoplasma
Commonly misidentified lines (See ICLAC register)	N/A

Animals and other research organisms

Policy information about studies involving animals; ARRIVE guidelines recommended for reporting animal research, and Sex and Gender in Research

Laboratory animals	C57BL/6 mice were purchased from GemPharmatech Co.,Ltd (Nanjing, China). Human CTLA-4 knockin mice were obtained from were obtained from the Shanghai Model Organisms Center, Inc. (Shanghai, China). 10-day-old and 7-8-week-old mice were used for experiments.
Wild animals	The study did not involve wild animals.
Reporting on sex	Both female and male mice were used in all the experiments.
Field-collected samples	No filled collected samples were used in the study.
Ethics oversight	All mice were maintained under specific pathogen-free conditions at the Tsinghua University. All studies were approved by the Animal Care and Use Committee of the Tsinghua University.

Note that full information on the approval of the study protocol must also be provided in the manuscript.

Flow Cytometry

Plots

Confirm that:

- [x] The axis labels state the marker and fluorochrome used (e.g. CD4-FITC).
- [x] The axis scales are clearly visible. Include numbers along axes only for bottom left plot of group (a 'group' is an analysis of identical markers).
- [x] All plots are contour plots with outliers or pseudocolor plots.
- [x] A numerical value for number of cells or percentage (with statistics) is provided.

Methodology

Sample preparation

For Flow cytometry: Tumor tissues were collected, cut into small pieces, and re-suspended in digestion buffer (RPMI-1640 medium with 1mg/mL type IV collagenase and 100μg/mL DNase I). Tumors were digested for 45 min at 37°C and then passed

	through a 70-µm cell strainer to make single-cell suspensions. Single-cell suspensions were incubated with CD16/32 blocking antibody and stained with specific antibodies followed by established protocol.
Instrument	Fortessa, FACSAria(BD™)
Software	BD FACSDiva™ Software was used for aquisition. FlowJow (V10) was used for analysis
Cell population abundance	After sorting, the sorted population was at least 90% pure as confirmed by further passage on another flow cytometer.
Gating strategy	The gated population is indicated in the Figure Legend or Methods.

☒ Tick this box to confirm that a figure exemplifying the gating strategy is provided in the Supplementary Information.
